# SpeedyPaddy: a revolutionized cost-effective protocol for large scale offseason advancement of rice germplasm

**DOI:** 10.1186/s13007-024-01235-x

**Published:** 2024-07-20

**Authors:** Nitika Sandhu, Jasneet Singh, Gomsie Pruthi, Vikas Kumar Verma, Om Prakash Raigar, Navtej Singh Bains, Parveen Chhuneja, Arvind Kumar

**Affiliations:** 1 Punjab Agricultural University, Ludhiana, Punjab 141004 India; 2 Delta Agrigenetics, Plot No. 99 & 100 Green Park Avenue, Village, Jeedimetla, Secunderabad, Telangana 500055 India

**Keywords:** Cost-effective, Germination, Light intensity, Light spectrum, Speed breeding, Nutrient, Photoperiod, Plant density, Rice

## Abstract

**Background:**

Improving the rate of genetic gain of cereal crop will rely on the accelerated crop breeding pipelines to allow rapid delivery of improved crop varieties. The laborious, time-consuming traditional breeding cycle, and the seasonal variations are the key factor restricting the breeder to develop new varieties. To address these issues, a revolutionized cost-effective speed breeding protocol for large-scale rice germplasm advancement is presented in the present study. The protocol emphasises on optimizing potting material, balancing the double-edged sword of limited nutritional dose, mode and stage of application, plant density, temperature, humidity, light spectrum, intensity, photoperiod, and hormonal regulation to accelerate rice growth and development.

**Results:**

The plant density of 700 plants/m^2^, cost-effective halogen tubes (B:G:R:FR-7.0:27.6:65.4:89.2) with an intensity of ∼ 750–800 µmol/m^2^/s and photoperiod of 13 h light and 11 h dark during seedling and vegetative stage and 8 h light and 16 h dark during reproductive stage had a significant effect (*P* < 0.05) on reducing the mean plant height, tillering, and inducing early flowering. Our results confirmed that one generation can be achieved within 68–75 days using the cost-effective SpeedyPaddy protocol resulting in 4–5 generations per year across different duration of rice varieties. The other applications include hybridization, trait-based phenotyping, and mapping of QTL/genes. The estimated cost to run one breeding cycle with plant capacity of 15,680 plants in SpeedyPaddy was $2941 including one-time miscellaneous cost which is much lower than the advanced controlled environment speed breeding facilities.

**Conclusion:**

The protocol offers a promising cost-effective solution with average saving of 2.0 to 2.6 months per breeding cycle with an integration of genomics-assisted selection, trait-based phenotyping, mapping of QTL/genes, marker development may accelerate the varietal development and release. This outstanding cost-effective break-through marks a significant leap in rice breeding addressing climate change and food security.

**Supplementary Information:**

The online version contains supplementary material available at 10.1186/s13007-024-01235-x.

## Background

The changing environment and growing population require significant increase in rice yields. The current rate of genetic gains in cereal crops is not adequate to meet the future demand. The seasonal variations and long generation time often limit to only 1–2 generation per year. The duration of seed-to-seed is one among the major bottlenecks in accelerating crop improvement. The traditional breeding practices used to develop new crop varieties are generally time-space-resource consuming and unable to keep up with the exponential rising demand for cereal production [2]. The advances in technologies enable the researchers and breeders to accelerate the advancement of novel crop varieties [5–7]. Atlin et al. [3] suggested that reducing the time of breeding cycle or accelerated breeding is one of the simplest ways to increase the genetic gain.

To address these limitations, various breeding techniques involving pedigree, bulk/modified bulk, single seed descent (SSD), shuttle breeding, doubled haploid (DH) and the rapid generation advancement (RGA) have been exploited in the self-pollinated crops to reduce the generation times and to foster the development of climate-resilient crops [16, 22, 24, 35]. Further, the speed breeding concept has been introduced to efficiently manage the environmental factors [15, 38]. Speed breeding mainly rely on temperature control, photoperiod extension, and the early seed harvest. It promotes the fast and accelerated growth and development from vegetative to reproductive stage in the high-density planting [8–10] reducing generation times and advancing multiple generations per year. The flexibility in speed breeding protocols allows it to line up and integrate with diverse research purposes including hybridization, phenotyping, population development, gene stacking, genomic selection, and genomic editing [29]. The plant breeding has produced many high-yielding crops that have allowed the growth of human population to continue for the past 100 years. The development of next-generation climate resilient high yielding varieties involving accelerated breeding will meet the requirement of population expansion in the coming decades.

The speed breeding technology has facilitated the rapid phenotyping in wheat and European barley [4, 17], introgression of useful allelic variation from the wild relatives in lentil [21], accelerated the development of herbicide-tolerant chickpea [12] and soyabean [19], and salinity tolerant rice [26]. The seed vernalization protocol in wheat and barley allowed five generations per year using speed breeding conditions [11] and six generations per year have been optimized in *Cannabis sativa* [32] and cassava (Rodrmguez et al. 2023 [28]) with different light conditions. These practical breeding outcomes highlight the potential of worldwide suite of the speed breeding techniques to substantially accelerate the crop genetic gain.

Although speed breeding is a powerful technique to increase the pace of genetic gain in crop species, it has several limitations. The major concerns include lack of the advanced controlled environment facilities, large scale multiplication of germplasm, stable supply of electricity and maintaining a comfortable temperature, and reliable water especially in the resource-poor nations with inadequate facility and infrastructures [10, 27]. For the establishment, regular proper maintenance, and smooth running of such high-cost facility; the developing countries need to invest more and more in plant breeding education, research, and the employee retention to support such long-term crop improvement projects and the scientific advancements [39]. It requires cutting-edge technologies, solid infrastructure to regulate environmental factors, which is a major limitation in many developing countries due to the limited institutional and government support, lack of facilities and specialized equipment. The cost can be reduced using ground-breaking local technologies with solar-powered light and temperature controls and semi-controlled field-based systems [29, 33]. The availability of a low-cost infrastructure highlights the versatility of the speed breeding, which can be custom-made according to the local resources and purposes.

Direct Seeded Rice (DSR) is a promising technology with water and labour-saving possibilities. The attributes required in a variety meant for direct seeding include anaerobic germination, ability of seed to emerge from depth, resistance/tolerance to bacterial late blight, brown plant hopper, brown spot, iron deficiency tolerance, weed competitiveness, tolerance to micronutrient deficiency, root plasticity for drought tolerance and nematode tolerance. However, limited genomics-assisted breeding efforts utilizing high throughput markers have been made for developing rice cultivars specifically for direct seeded conditions. Considering this, the present study aims to present a cost-effective facility for large scale advancement and off-season affordable phenotyping of rice germplasm which has served well in the development and validation of SNP based Kompetitive Allele Specific Polymorphism (KASP) assays and in the introgression and development of inbred lines containing important direct seeded traits such as anaerobic germination and deeper sowing depth.

## Materials and methods

### Transformed local infrastructure for SpeedyPaddy

The SpeedyPaddy protocol was standardized at School of Agricultural Biotechnology, PAU (30 ° 54’ N latitude, 75 ° 48’ E longitude, and 247 m above sea level). Punjab is situated in north-eastern part of India and experiences extreme hot and cold weather conditions. The average temperature in winter season ranges 7 to 15 °C maximum to 0 to 8 °C minimum. The average relative humidity in winter season remains 70%.

The existing 67.16 m^2^ fiber sheet screenhouse facility with basic water and power supply was used to standardized the low-cost common man speed breeding protocol. The existing facility is equipped with twenty units of low-cost halogen based 500-watts light tubes, two 1500-watt capacity coiled heaters and one greenhouse humidifier (Eurotemp, model no: HW-26MC03).

### Plant material

The protocol was standardized using early (PR126, PB1509), medium (PR121, PR128, PR129), late duration (Swarna, Samba Mahsuri) rice varieties in 2018–2019. The standardized protocol was validated on segregating generations including F_2_, F_3_, F_4_ generations and advanced breeding lines in 2019–2020, and 2020–2021. The validated protocol is being utilized since 2021 for hybridization, generation advancement, trait based-phenotyping, marker-assisted selection, and development of homozygous improved advanced breeding lines.

### Prerequisites for rice plant establishment

A total of 16 portable aluminum benches of 2.0 m length and 1.2 m width each were used to place 160 plastic crates of 40 cm width, 60 cm length and 7 cm height each. The 21 wells (21″x11″x3.25″), 50 wells (21″x11″x2.0″) and 98 wells (21″x11″x1.5″) plug trays and pots, (volume: 490.2 cm^3^) were used for establishment of rice plants.

### Optimization of protocol for SpeedyPaddy

Major requirements for the speed breeding include efficient utilization of space, regulated temperature, humidity, and light source.

### Optimization of rice germination

Early and uniform germination serves as an important pillar for rice plant establishment. We tested eight different combinations of soil, farmyard manure and cocopeat while keeping the other parameters constant to standardize the best combination for early germination.

### Optimizing plant density for growth characteristics

The plant density at spacing of 1 plants/0.14m^2^ in pots, 21 plants/0.14m^2^, 50 plants/0.14m^2^, and 98 plants/0.14m^2^ in plug trays and was tested. The effect of plant density on vegetative growth, tiller number, plant height and maturity were studied.

### Optimization of nutrient composition and application

Different combinations of nutritional doses, the mode, type, and stages of application were studied (Supplementary Table 1). Two different sets of standardization experiments were conducted with three treatments in each set. In the first set, the mode and type of nutritional doses were standardized. Building on the results, the second set of experiment was conducted to identify the exact stage of nutrient application.

### Optimization of light source, photoperiod, and temperature

The effect of light source and photoperiod was studied to select the high resource efficient and low-cost set-up. The light source includes natural sunlight, full spectrum light bulbs, and low-cost halogen based 500-watts light tubes. Two different photoperiods at different stages of plant growth and development were tested for 500-watts light tubes (Supplementary Table 2). The full spectrum light bulbs and the halogen based 500-watts light tubes maintained the PPFD of ∼ 750–800 µmol/m^2^/s at plant canopy height and 30–32 °C temperature during day hours. During night hours the 23 to 25 °C temperature was regulated using two 1500-watt capacity coiled heaters.

### Tuning and tweaking of early seed harvest

To reduce the crop duration, the prematurely harvested seeds after 10, 12, 15 days of anthesis were kept in an incubator at 38 °C for the conversion from milky to the maturation stage for 24 h. Dry seeds were treated with 100 ppm of GA_3_, 2% CaCl_2_, 1% KH_2_PO_4_ and 40 ppm Na_2_SeO_3_ at 16 to 18 °C for 20 h. The treated seeds were washed 2–3 times with distilled water and sown directly into the plug trays for the next cycle of SpeedyPaddy.

### Validation of SpeedyPaddy protocol

After standardization, the validation of the SpeedyPaddy protocol was conducted at different levels. The SpeedyPaddy protocol was used for the advancement of segregating generations, hybridization, trait-based phenotyping including screening for anaerobic germination and germination from deep sowing depth and raising of populations for mapping of QTL/genes.

## Results

### Optimization of protocol for SpeedyPaddy

The four different experiments were conducted to optimize the protocol for SpedyPaddy. Each experiment was conducted keeping one variable factor and other constant factors. The optimization includes (i) early and uniform germination, (ii) plant density for growth characteristics, (iii) nutrient composition, mode, and stages of nutrient application, and (iv) light source, temperature, and photoperiod.

**Fig. 1:**
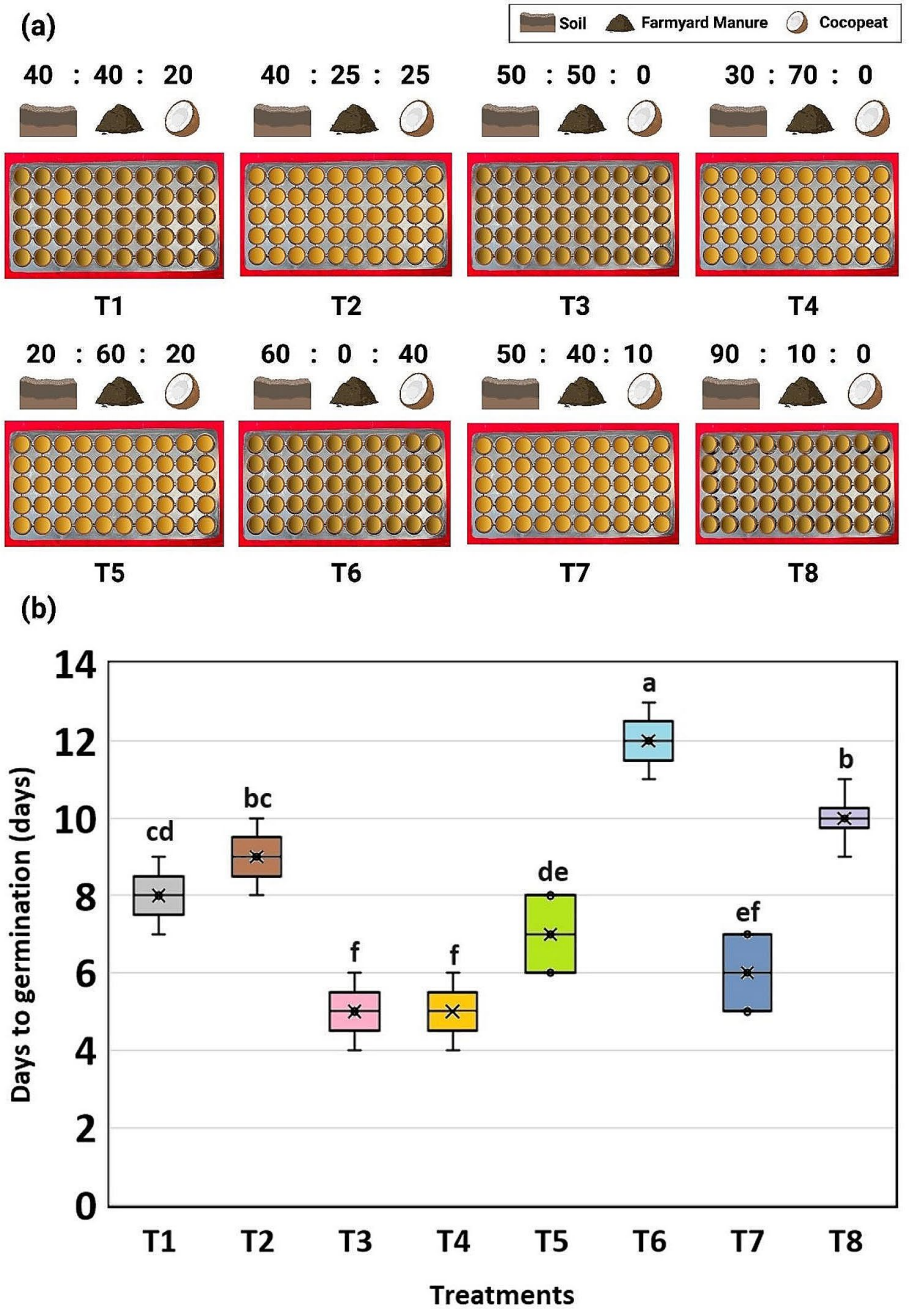
The graph illustrates the effects of eight different treatments on the average days to germination. The mean days to germination of the 7 different varieties {early (PR126, PB1509), medium (PR121, PR128, PR129), late duration (Swarna, Samba Mahsuri) was calculated and the mean days to germination were evaluated on the Y-axis. While eight different combinations of soil, farmyard manure and cocopeat are plotted on the X-axis using the box plots. For the box plots, the boxes denote 25th to 75th percentile, whiskers denote full data range, and the cross symbols denote the mean and center lines denote the median. The alphabets shown above the boxes (a, b, c, d, e, and f) represent statistical significance between the different treatments computed using Tukey’s test (*P* < 0.05)

First, we have standardized the rice germination. To check the early and fast germination, eight treatments involving different combinations of soil (S), farmyard manure (FYM) and cocopeat (C) were tested on 7 different varieties {early (PR126, PB1509), medium (PR121, PR128, PR129), late duration (Swarna, Samba Mahsuri)} used in the present study (Fig. 1a, Supplementary Table 3). The average value of early and uniform germination across varieties of different durations was calculated. The early and uniform germination at 5 days after sowing (DAS) was observed in treatment 3 (S-50%: FYM-50) and treatment 4 (S-30%: FYM:70) followed by 6 DAS in treatment 7 (S-50: FYM-40: C-10) and 7 days after sowing in treatment 5 (S-20: FYM-60: C-20). The very late germination 12 DAS was observed in treatment 6 (S-60: FYM-0: C-40). All the eight different treatments had a significant effect (*P* < 0.05) on rice germination (Fig. 1b).

**Fig. 2:**
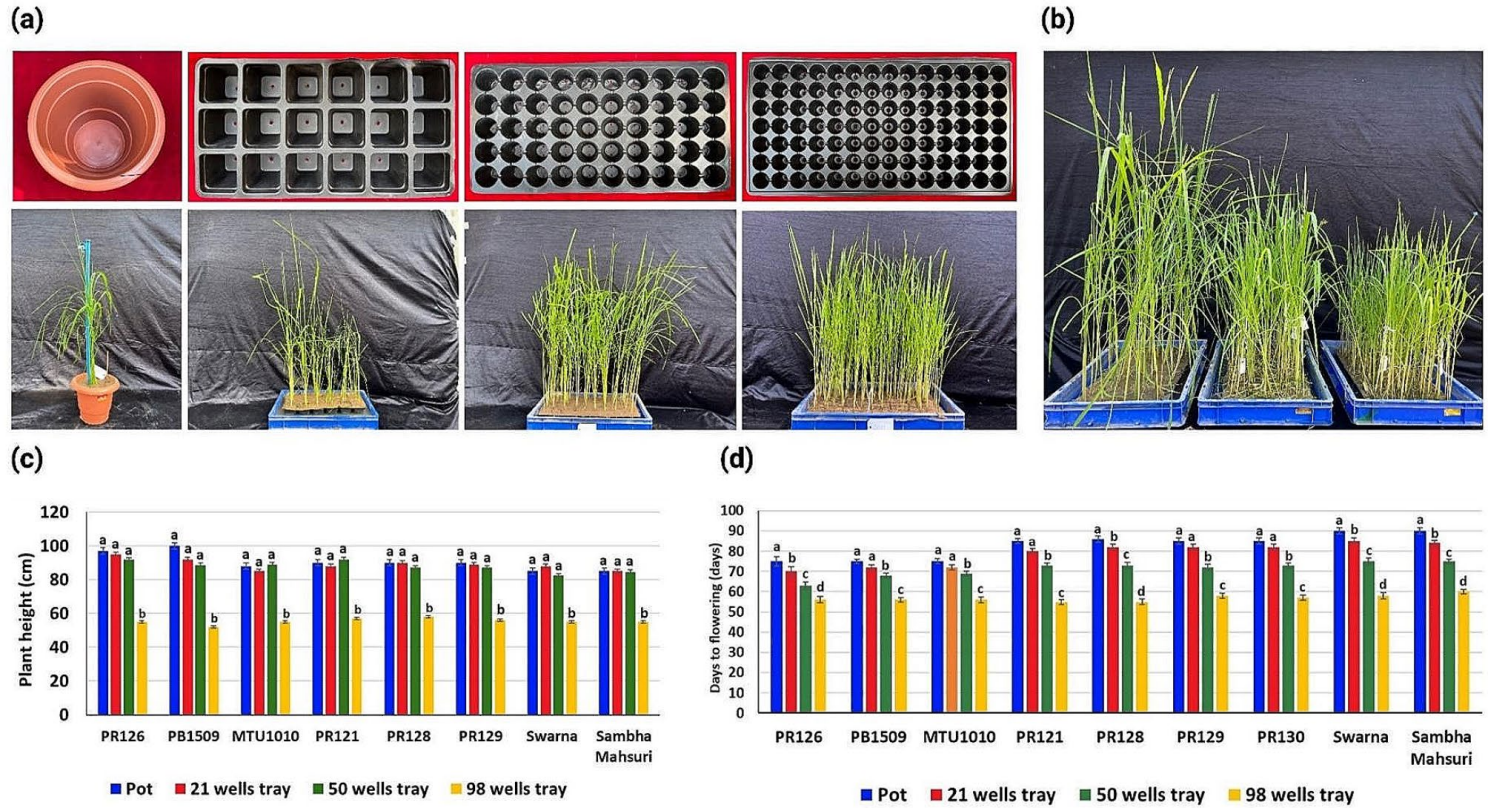
Optimization of SpeedyPaddy protocol for plant density for growth characteristics (**a**) The four different types of potting material tested to study the effect of plant density on plant height and days to flowering. The plant density of 1 plants/0.14m^2^ in pots, 21 plants/0.14m^2^ in 21 wells plug tray, 50 plants/0.14m^2^ in 50 wells plug tray, and 98 plants/0.14m^2^ in 98 well plug trays was tested (**b**) The comparison of plant height in 21, 50 and 98 wells plug tray with plant density of 21, 50 and 98 plants in 0.14m^2^, respectively. (**c**) The bar graphs representing the effect of four different plant densities on the mean value of plant height of different varieties tested in the present study. The alphabets above the bar graphs (**a** and **b**) designate the statistical significance between different plant densities groups of eight different varieties computed using Tukey’s test (*P* < 0.05). The different plant densities are presented on X-axis and the plant height of different varieties are shown on the Y-axis (**d**) The bar graphs representing the effect of four different plant densities on the mean value of days to flowering of different varieties of variable duration (early duration: PR126, PB1509 and MTU1010; medium duration: PR121, PR128, PR129; late duration: Swarna, Samba Mahsuri) tested in the present study. The alphabets above the bar graphs (a, b, c, and d) designate the statistical significance between different plant densities groups of eight different varieties computed using Tukey’s test (*P* < 0.05)

Secondly, we examined the effect of four different plant densities. The plant density of 4 plants/0.14m^2^ in pots, 21 plants/0.14m^2^ in 21 wells tray, 50 plants/0.14m^2^ in 50 wells tray, and 98 plants/0.14m^2^ in 98 well plug trays was tested (Fig. 2a). The effect of plant density on vegetative growth, tiller number, plant height and days to flowering was studied. The number of tillers in pots ranged from 2 to 5 tillers/plant, in 21 well trays ranged from 2 to 3 tillers/plant, in 50 wells plug trays from 1 to 2 tillers/plant, and single tiller in 98 wells tray. The plant density had significant effect (*P* < 0.05) on plant height (Fig. 2b and c) and days to flowering (Fig. 2d). The plant height in pots, 21 wells, 50 wells and 98 well trays varied from 85 to 100 cm, 85 to 95 cm, 82 to 92 cm, and 52 to 58 cm, respectively (Supplementary Table 4). The average days to flowering in early duration varieties was 75 days in pots, 71 days in 21 well trays, 67 days in 50 well trays and 56 days in 98 well trays (Supplementary Table 5).

**Fig. 3:**
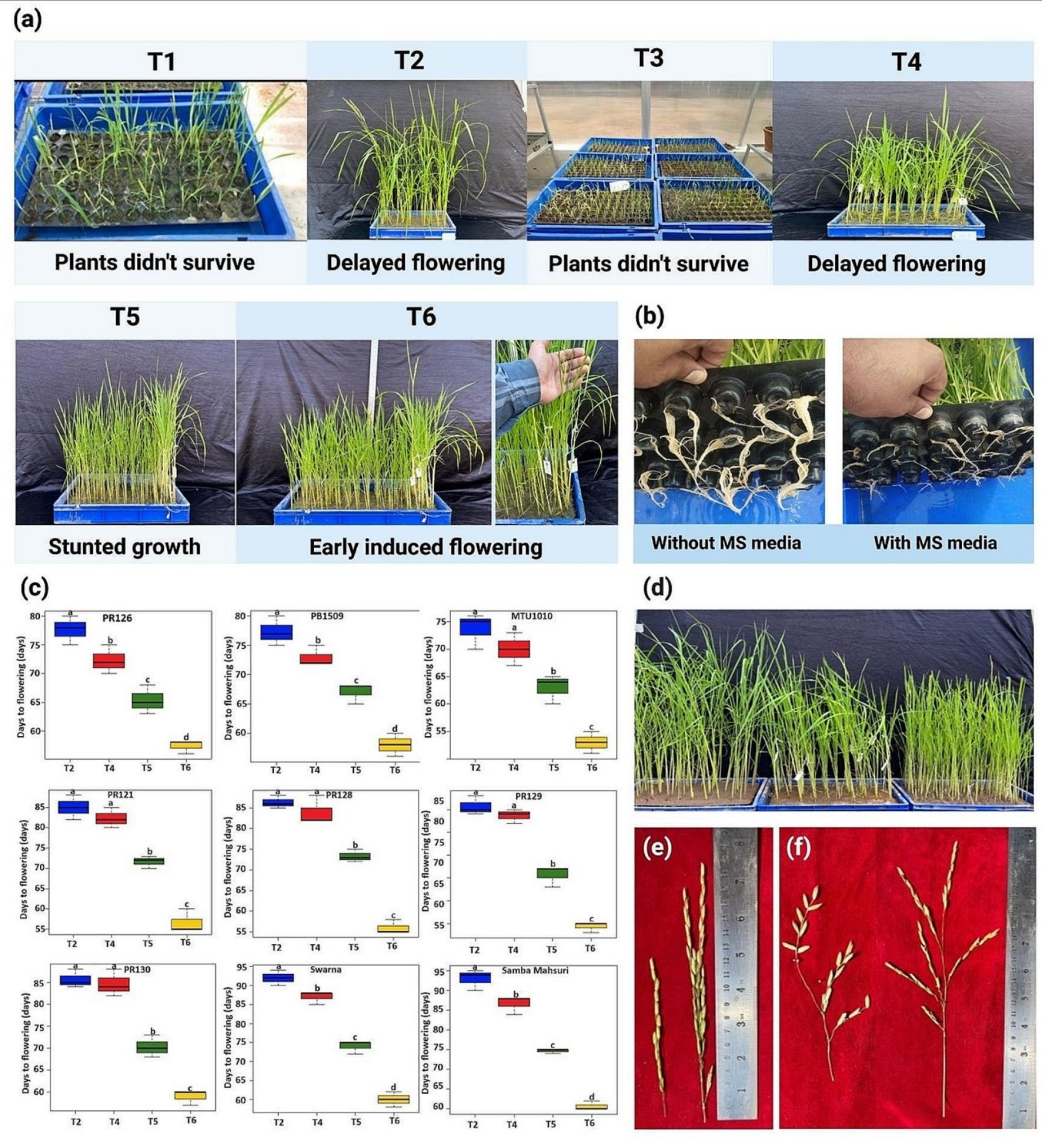
Optimization of SpeedyPaddy protocol for composition of nutrients, mode, and stages of application of nutrients (**a**) Effect of six treatments involving different mode of nutrient application, different combinations of nutrients, and application of nutrient at different stages of growth and development. The alphabet T designates the treatment and number designate the treatment number. T1: treatment 1 designate the foliar application of 1% NPK at 7th day, 1% FeSO_4_ at 15th day, 1% Zn nutrients at 18th day, 1% FeSO_4_ at 22th day, 1% NPK and 1% Zn at 32th day and 1% NPK at 40th day of growth cycle; T2: treatment 2 designate the application of 1% NPK at 7th day, 1% FeSO_4_ at 15th day, 1% Zn nutrients at 18th day, 1% FeSO_4_ at 22th day, 1% NPK and 1% Zn at 32th day and 1% NPK at 40th day of growth cycle via fertigation; T3: treatment 3 designate the application of 5% MS basal media at 7th day, 10% MS basal media at 15th day, 5% MS basal media at 22th day, and 10% MS basal media at 32th day of growth cycle via fertigation, T4: treatment 4 designate the application of 1% NPK at 7th day, 1% Zn at 15th day, 1% NPK at 22th day, 1% Zn at 28th day, 1% NPK at 32th day, and 1% NPK at 45th day of growth cycle via fertigation; T5: treatment 5 designate the application of 0.5% NPK at 7th day, 0.5% Zn at 15th day, 0.5% NPK at 18th day, 1% NPK at 28th day, and 1% NPK at 45th day of growth cycle via fertigation; T6: treatment 6 designate the application of 0.5% NPK at 7th day, 0.5% Zn at 15th day, 0.5% NPK at 18th day, and 1% NPK at 28th day of growth cycle via fertigation. The pictorial representation of root system represents the effect of MS media on the root system and the without MS media represents the root system in treatment (T6) (**b**) The graph illustrates the effects of four different treatments (T2, T4, T5 and T6) on the mean days to flowering of different varieties of variable duration (early duration: PR126, PB1509 and MTU1010; medium duration: PR121, PR128, PR129; late duration: Swarna, Samba Mahsuri) tested in the present study. The mean days to flowering were evaluated on the Y-axis. While four different treatments are plotted on the X-axis using the box plots. The plants in the treatments T1 and T3 did not survive. For the box plots, the boxes denote 25th to 75th percentile, whiskers denote full data range, and the center lines denote the median. The alphabets shown above the boxes (a, b, c, and d) represent statistical significance between the different treatments computed using Tukey’s test (*P* < 0.05). (**c**) The comparison of plant height in treatments T4, T5 and T6 (**d**) The effect of treatments T6 (left) and T4 (right) on panicle length, and (**e**) The effect of treatments T6 (left) and T4 (right) on grain number

At the third step of standardization of SpeedyPaddy protocol, we examined the effect of six different combinations of nutritional doses, the mode, and stages of nutrient application (Supplementary Table 1). At the first step, the mode and type of nutritional doses were standardized followed by the experiment to identify the exact stage of nutrient application. It is important to maintain the optimal nutritional stress to reduce vegetative growth and avoid plant death. The water was constantly provided in the trays to avoid any kind of drought stress during vegetative growth. The application of nutrient was found to be more effective by solubilizing it in tray water rather than the foliar spray (Fig. 3a). The phytotoxicity was observed in the treatment involving foliar spray. The solubilized application ensured slow and long-term effect of nutritional dose (Fig. 3a). The induction of nutritional stress before the seedling establishment turned out to be lethal and led to high plant death. The effect of gradual decrease of nutritional dose at seedling and vegetative stage was studied. The application of nutrients in form of basal MS media showed negative impact on root system. The root growth was highly reduced in the treatment involving application of nutrients via MS media compared to the treatment involving nutrient application in form of NPK and Zn (Fig. 3b). The application of nutrients in treatment 4 was in same manner as that of field conditions, whereas in treatment 5, the stages of nutrient application was kept same as field conditions, but the dose was reduced to half at seedling growth stage (Supplementary Table 1). Only three nutrient dose was applied in treatment 6 compared to four nutrient doses in treatment 5 (Supplementary Table 1). The average plant height in treatment 2, 4, 5 and 6 was 92, 91, 66 days and 55 cm, respectively (Supplementary Table 6). The average days to flowering in treatment 2, 4, 5 and 6 was 85, 81, 71 days and 58 days, respectively (Supplementary Table 7). The analysis revealed a significant difference among the treatment groups in terms of reducing the flowering time (*P* < 0.05) (Fig. 3c). The nutrient application in form of 0.5% NPK (14N:14P:14K) at 7–8 days and 18th day of seedling growth stage, 0.5% Zn at 15th day of seedling growth and 1% NPK at 28th day of vegetative growth showed significant effect on plant height (Fig. 3d), panicle length (Fig. 3e) and grain number (Fig. 3f).

**Fig. 4:**
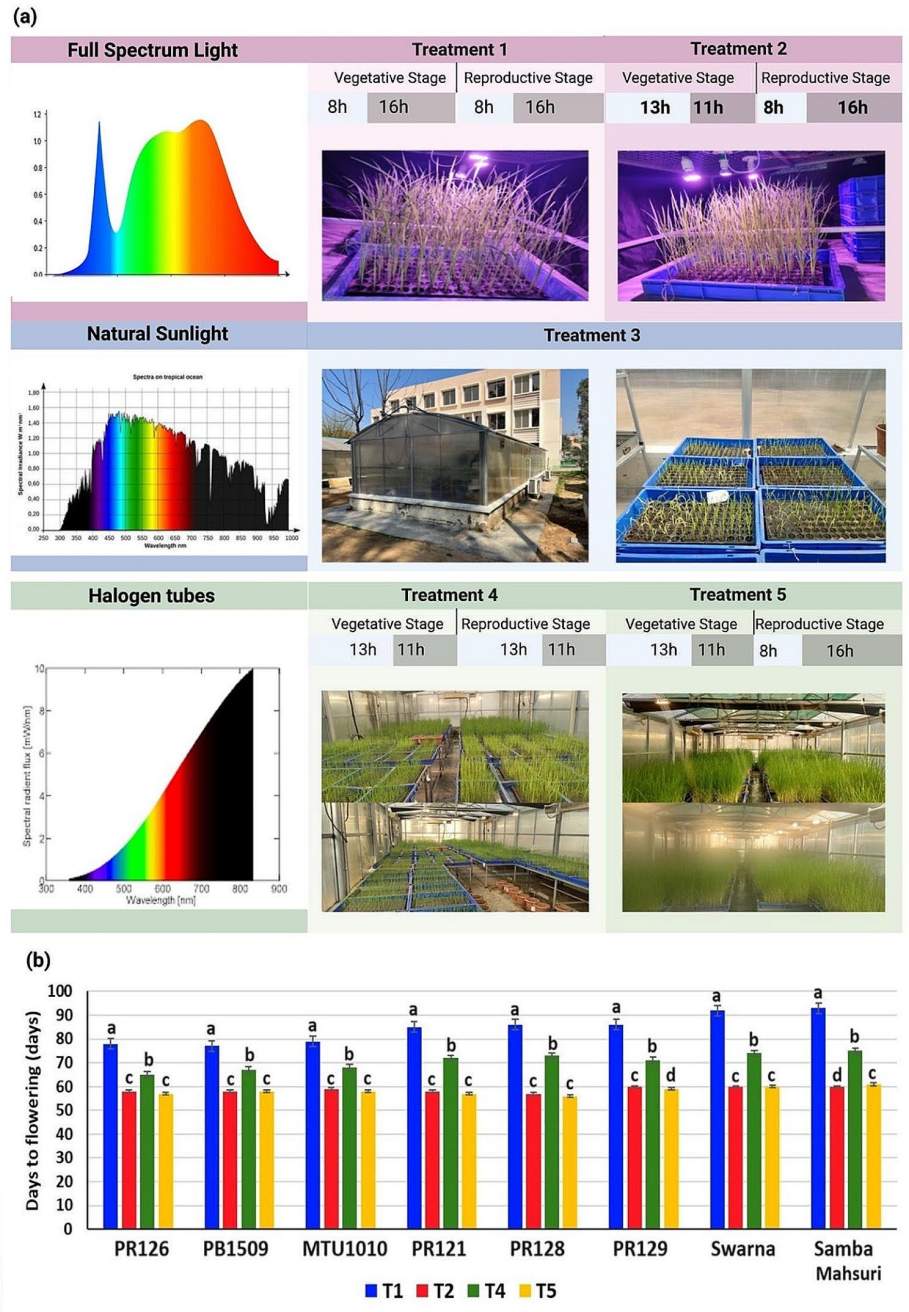
Optimization of SpeedyPaddy protocol for light source and photoperiod. (**a**) The picture illustrates five treatments involving different source of light and photoperiod. The alphabet T designates the treatment and number designate the treatment number. T1: treatment 1 designate the full spectrum artificial light (PPFD of ∼ 750–800 µmol/m^2^/s, light/dark: 8 h/16 h at seedling, vegetative and reproductive stages); T2: treatment 2 designate the full spectrum artificial light (PPFD of ∼ 750–800 µmol/m^2^/s, light/dark: 13 h/11 h at seedling and vegetative and 8 h/16 h at reproductive stages; T3: treatment 3 designate the natural spectrum/no artificial light; T4: treatment 4 designate the halogen bulbs (B:G:R:FR-7.0:27.6:65.4:89.2, light/dark: 13 h/11 h at seedling, vegetative and reproductive stages); T5: treatment 5 designate the full spectrum artificial light (halogen bulbs (B:G:R:FR -7.0:27.6:65.4:89.2, light/dark: 13 h/11 h at seedling and vegetative and 8 h/16 h at reproductive stages) (**b**) A statistical comparison shows the difference between mean days to flowering of different varieties of variable duration (early duration: PR126, PB1509 and MTU1010; medium duration: PR121, PR128, PR129; late duration: Swarna, Samba Mahsuri) which was used for protocol optimization in SpeedyPaddy. The alphabets above the bar graphs (a, b, c, and d) designate the statistical significance between treatments T1, T2, T4, and T5 of eight different varieties computed using Tukey’s test (*P* < 0.05)

We have conducted five distinct experiments to study the effect of light source and photoperiod (Fig. 4a). The light source includes natural sunlight, full spectrum light bulbs, and low-cost halogen based 500-watts light tubes. Different photoperiod and source of light had varying effect on flowering. The full spectrum artificial light and halogen based 500-watts light tubes maintaining the B:G:R:FR - 7.0:27.6:65.4:89.2, and an intensity of ∼ 750–800 µmol/m^2^/s at plant canopy height with photoperiod of 13 h light and 11 h dark during seedling and vegetative stage and 8 h light and 16 h dark during reproductive stage had a significant effect (*P* < 0.05) on reducing the mean flowering time among tested rice varieties as compared to field or natural sunlight spectrum (Supplementary Table 8), Fig. 4b. The high-cost full spectrum artificial light and cost-effective halogen bulb with variations in the photoperiod showed similar results. The night hours temperature between 23 and 25°C was regulated using two 300-watts capacity coiled heaters.

### Premature seed harvesting and hormonal treatment reduce maturity time

**Fig. 5:**
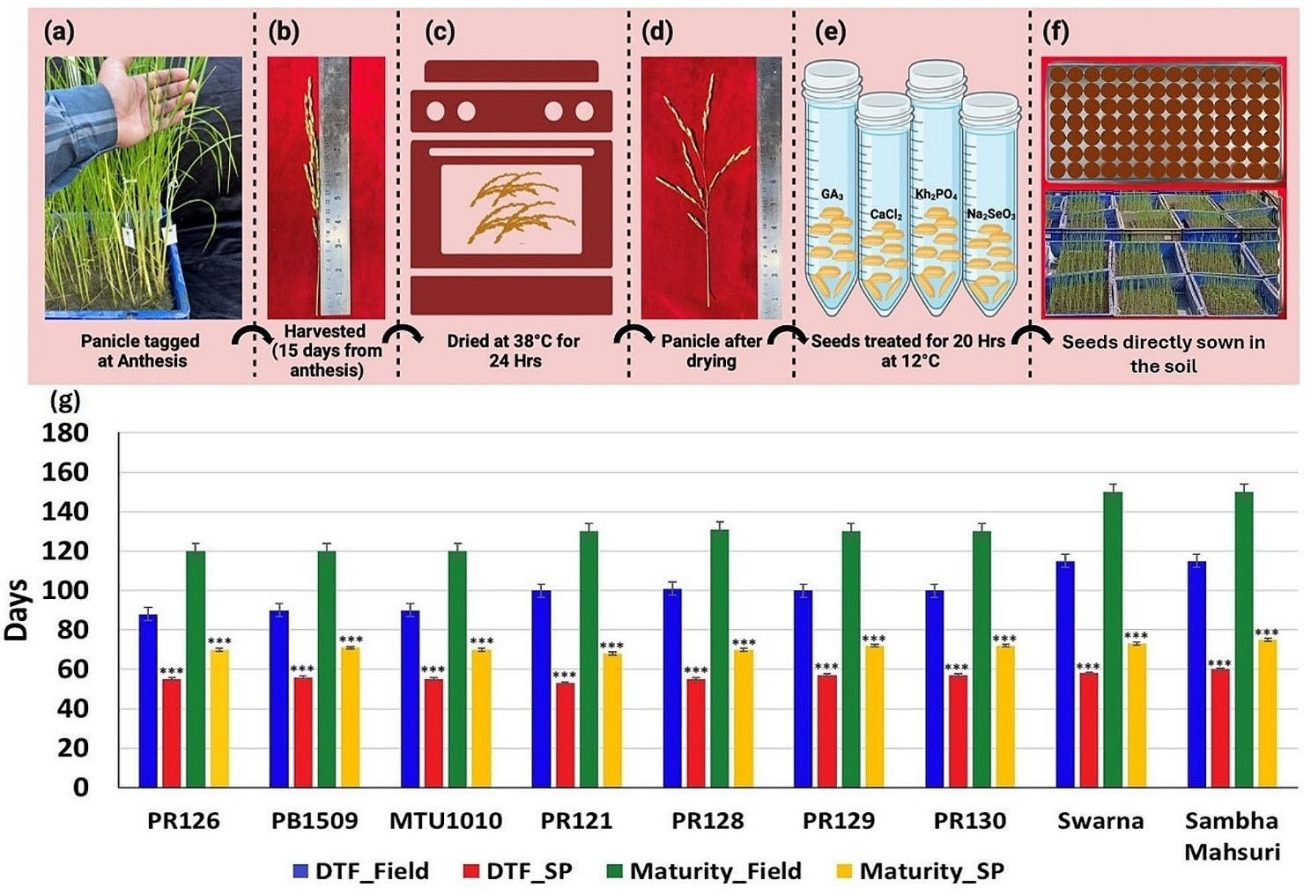
Optimization of SpeedyPaddy protocol for premature seed harvest and germination (**a**) The picture illustrates the tagging of panicles at anthesis (**b**) Prematurely harvested panicle at 15 days after anthesis (**c**) Drying of prematurely harvested seeds at 38 °C for 24 h (**d**) dried panicle (**e**) treatment of dried seed with GA_3_, CaCl_2,_ KH_2_PO_4_ and Na_2_SeO_3_ for 20 h at 12 °C. (**g**) The statistical comparison shows the difference between mean days to flowering in the field and SpeedyPaddy in eight varieties of different duration (early duration: PR126, PB1509 and MTU1010; medium duration: PR121, PR128, PR129; late duration: Swarna, Samba Mahsuri), which was used for the optimization of protocol in SpeedyPaddy. The statistical significance was determined by the t-test: ***P < 0.001

To accelerate the seed maturity and reduce the total crop time in SpeedyPaddy, the panicles of rice plants were tagged on the day of anthesis and harvested prematurely at early dough stage 15 days after anthesis (Fig. 5a and b). The seeds were dried at 38°C for 24 h for maturation and dormancy breakage (Fig. 5c and d). Following this, four different germination agents including GA_3_, CaCl_2_, KH_2_PO_4,_ and Na_2_SeO_3_ were tested to check their effect on inducting early and uniform germination (Fig. 5e). The treated seeds were directly sown to the plug trays containing soil and farmyard manure (Fig. 5f). Out of these, the treatments containing 100 ppm of GA_3_ and 2% CaCl_2_ were most effective with 70% and 65% germination induction, respectively. The lowest germination percentage of 20% was observed in the seeds treated with KH_2_PO_4_ (Supplementary Table 10).

### Validation of SpeedyPaddy protocol

Upon standardization and inclusive analysis of each parameter pertaining to germination, nutrient dose and application, induction of flowering and early maturation; the parameters that showed significant effect on all the tested varieties were compiled to formulate a final SpeedyPaddy protocol. The final protocol was then validated on different sets for the advancement of segregating generations, hybridization, trait-based phenotyping including screening for anaerobic germination and germination from deep sowing depth and raising of populations for mapping of QTL/genes. Overall, the SpeedyPaddy protocol resulted in average saving of 51, 60 and 76 days in the early, medium, and late duration tested rice varieties, respectively (Fig. 5g). The SpeedyPaddy protocol enables the possibility to advance 4–5 generations based on their maturity duration (Supplementary Table 11).

**Fig. 6:**
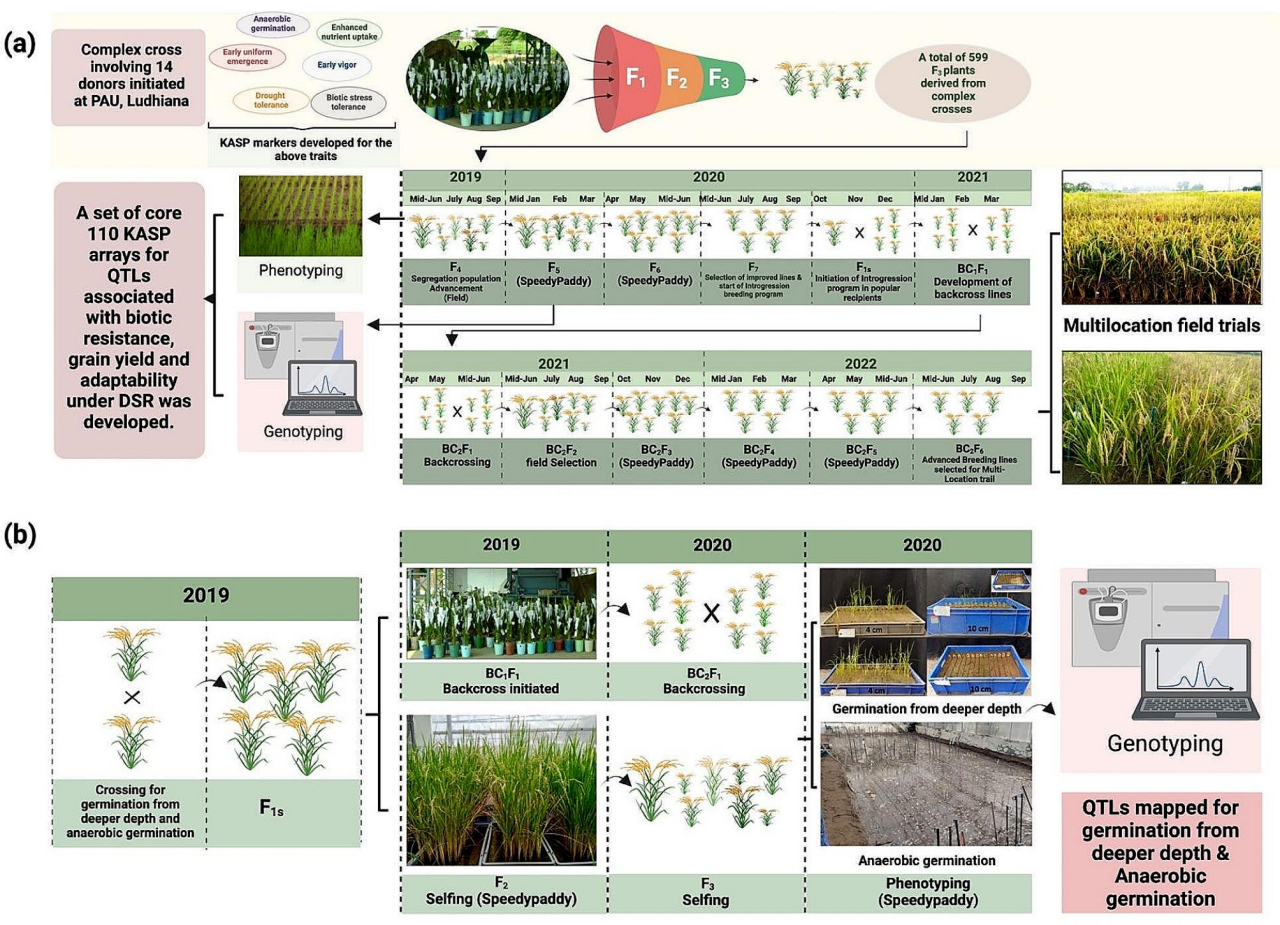
The presented illustration showed the validation of SpeedyPaddy protocol on different sets (**a**) advancement of segregating generations, hybridization, selfing, backcrossing and genomics-assisted introgression (**b**) hybridization, trait-based phenotyping including screening for anaerobic germination and germination from deep sowing depth and raising of populations for mapping of QTL/genes

A subset of 599 segregating breeding lines at F_4_ generation developed through forward breeding approach involving 14 different donors were advanced to F_5_ and F_6_ in 2019–2020 in SpeedyPaddy facility (Fig. 6a). After field evaluation of the above-mentioned breeding material in 2020 *kharif* season, a subset involving 10 promising breeding lines was selected and hybridization program for introgression of QTL/genes associated with traits improving adaptation of rice under DSR in background of PR126 and PB1509 was initiated in October 2020 in SpeedyPaddy facility. The F_1_ seeds were harvested and backcrossed in January 2021. A total of 20 panicles per background was used to cross with each donor. The seed set of F_1_ seed ranged from 5 to 8 seeds per panicle. An average of 70 to 75 F_1_ seeds per cross was generated and 45 to 50 seeds per cross survived after germination. These seeds were then backcrossed and the backcross seeds were advanced in April-June 2021 and BC_1_F_2_ were evaluated in 2021 *kharif* season under field conditions. The plant selection was carried out under field conditions and selected lines were advanced to BC_1_F_4_, BC_1_F_5_ and BC_1_F_6_ in SpeedyPaddy in 2021–2022. The breeding material was evaluated and seed of selected lines was multiplied under field conditions in 2022 *kharif* season. The breeding material reached to multilocation evaluation in just two years because of SpeedyPaddy which generally takes 6 to 7 years under traditional method. Another set including 42 advanced breeding lines possessing different QTL/genes associated with traits improving adaptability of rice under DSR [31] were procured from International Rice Research Institute (IRRI), Philippines in 2019 and few seeds were planted in offseason to be used for validation for the developed KASP assays ([30], Fig. 6a). In an experiment of trait-based phenotyping, a total of 1500 segregating breeding lines developed involving donors for anaerobic germination and germination from deep sowing depth were phenotyped in 2023–2024 in the same facility (Fig. 6b). The breeding lines possessing the ability to germinate under anaerobic conditions as well as capability to germinate from deep sowing depth were selected for further field evaluation. For the experiment involving mapping of genomic regions associated with germination from deep sowing depth, the F_1_ seeds of PR126/IRGC 128,442 were produced in field in 2019 kharif season. The F_1_ seeds were advanced to F_2_ and F_1_ seeds were also backcrossed to PR126 in 2019–2020 rabi season under speedpaddy conditions. The F_2_s were advanced to F_3_ and the BC_1_F_1_s were backcrossed to PR126 to generate BC_2_F_1_s. The mapping and fine mapping was conducted on F_3:4_ and BC_2_F_2:3_.

### Cost-effectiveness of SpeedyPaddy

The total estimated cost per breeding cycle of 3 months includes cost of heaters, halogen bulbs, humidifier, plastic crates, plug trays, nutrients, consumption of electricity {(heaters: 2 (1500 watts each; halogen bulbs: 20 (500 watts each), humidifier (260 watts)}, labor and maintenance cost. The estimated cost to run one breeding cycle in SpeedyPaddy was $2941 including one time cost of plastic crates which is much lower than the advanced controlled environment speed breeding facilities (Supplementary Table 9). The existing plant capacity of the fiber sheet screenhouse facility, SpeedyPaddy of 67.16 m^2^ was 15,680 plants.

## Discussion

Traditional breeding for novel and improved cultivars with market-preferred traits generally take more than 10 years in the absence of any technological interventional in the pre-breeding programme [1, 13]. In early era of breeding program, a significant amount of space, time, and resources were devoted in the selection and the genetic advancement stages. Depending upon the distinct groups of *Oryza sativa* L., the vegetative stage in rice ranged from 30 to 90 days, while the reproductive to maturation stage ranged from 30 to 40 days [40]. To accelerate the generation advancement and to reduce the duration of breeding cycle, each growth stage must be shortened. Therefore, aiming to save time through rapid generation advancement, speed breeding involves manipulation of the crop growing environment provides an opportunity to advance the next breeding generation as quickly as possible [14, 18].

The optimization of protocol for long-day plants involves standardization of nutrient stress, plant density, growth stage and growth parameters to achieve fast growth, early flowering, and fast maturation [26]. Generally, a mixture of sand, cocopeat and farmyard manure is recommended as potting material for the crop species. In contrast, in our present study the treatments containing cocopeat did not show good germination. This might be probably due to low water retention, high potassium content of cocopeat and high pH. Our experimental results demonstrated that combination of soil and farm yard manure in ratio of S - 50%: FYM - 50 and S - 30%: FYM - 70% and covering of trays with clear plastic sheet for initial 5 days significantly improved germination. The clear plastic sheet may provide the best covering *via* keeping check for hydration and germination. After the establishment of rice seedling, the standing water in the plastic crate until reproductive stage had shown to support plant growth. The high planting density is one among the low-cost speed breeding strategies appropriate for the rapid advancement of generations, while maintaining large population size required for the advanced selections. Our results revealed that the plant density of 98 plants/0.14m^2^ or 700 plants/m^2^ was effective in reducing plant height, tillering, and inducing early flowering. The spatial stress worked excellently to induce stunted growth and low tillering. Low space induced competition among plants, caused zero tillering, reduce weed stress, and helped increase the number of plants in the screenhouse. In rice, up to four generations/year and shortening of breeding cycle by 15 to 40 days were achieved with a high-density planting of 400 plants/m^2^ [25].

Nutrients are very crucial for the growth and development of plant. The nutrient deficiencies and imbalances at different stages of growth and development and mode of nutrient application can adversely affect the plant growth, development, and maturity. In our study, we found that the limited application of nutrients at seedling and vegetative stage had a significant impact on maintaining the plant biomass, height, and induction of early flowering. Interestingly, we observed that restricting nitrogen application after stem elongation or flag leaf emergence promoted early flowering in rice. The negative impact of MS media was observed on root system. Similarly, slower primary root growth was observed in *Arabidopsis thaliana* after application of MS media [20, 36, 37]. The application of NPK and Zinc was found to be more effective by solubilizing it in tray water rather than the foliar spray. The solubilized application ensured slow and long-term effect of nutritional dose.

Although rice is non-sensitive to varying photoperiods, the different types of light systems including halogen lights, full spectrum lights and role of photoperiod in achieving synchronized and early flowering were studied to find the cost-effective system. Mimicking the natural photoperiod trend of reduced exposure after the tillering stage resulted in early flowering and reduced plant height. The proper understanding of photoperiod-sensitive and photoperiod-insensitive phases in rice is required for inducing early flowering. Previous studies showed the role of extended photoperiods in inducing early flowering in both the short-day and long-day plants [11, 15, 26, 38]. The plants exposed to photoperiods of 13 h light and 11 h dark for the initial seedling and vegetative stage, followed by an instant shift to the short-day conditions of 8 h light and 16 h dark demonstrated significant effect on inducing early flowering. This highlights the importance of photoperiod duration during the specific seedling and vegetative stages in achieving uniform and early flowering in rice. With the cutting-edge variable light spectrums, the quality, potency, flavour, yield and accelerated varietal development can be achieved. Interestingly, in our present study the plants were extremely sensitive to the difference between red and blue spectrum, which should not be interpreted as exclusion of other spectrums. The balance between the two (high red-to-blue spectrum ratio (2R > 1B) using low-cost halogen lights (B: G:R:FR-7.0:27.6:65.4:89.2) only can give rice crop very precise instructions on how to grow. Further, we maintained 30–32°C temperature during day hours and 23 to 25°C temperature during night hours and humidity of 70% throughout the experiments. However, it is worth mentioning that although the balance between temperature and humidity facilitated the early flowering, but reduced plant height, panicle length, grain size, grain number and both filled and chaffy grains were observed. It indicates the impact of stress conditions on seed development and grain quality. Significant variations were observed in the flowering and maturity of the tested varieties in SpeedyPaddy compared to the field conditions. Overall, the SpeedyPaddy protocol resulted in average saving of 51, 60 and 76 days in the early, medium, and late duration tested rice varieties, respectively (Supplementary Table 11). Importantly, our optimized Speedypaddy parameters yielded positive results in late duration varieties, such as Swarna and Samba Mahsuri, which typically takes 150 days to flower in the field but only 58 to 60 days in the Speedypaddy facility. These findings highlight the importance of manipulating the photoperiod duration during specific stages to accelerate flowering in rice. Furthermore, the protocol provides accurate knowledge about the transition of rice crop from the photoperiod-insensitive to the photoperiod-sensitive phase via provision of extended daylight exposure, leading to the significant saving of crop development time.

Speed breeding for any crop requires an inclusive understanding of its physiological growth stages. Although, speed breeding is a valuable approach to fast-track the conventional breeding programme, but it requires technical expertise, effective, modern, and complementary plant phenomics facilities, adequate infrastructure and continuous financial support for the research and development activities [23, 34]. The innovative solutions such as the small indoor speed breeding kit with fitted LED lights, humidity and temperature controls powered by the solar system and the semi-controlled field-based systems with high-dense planting, temperature-humidity controller, and nutrient stress could be developed using the existing technologies. In the present study, we have exploited the available resources to achieve target of 4 to 5 rice generations per year with a minimum cost of ∼ US$ 2623/breeding cycle including some one-time miscellaneous cost.

**Fig. 7:**
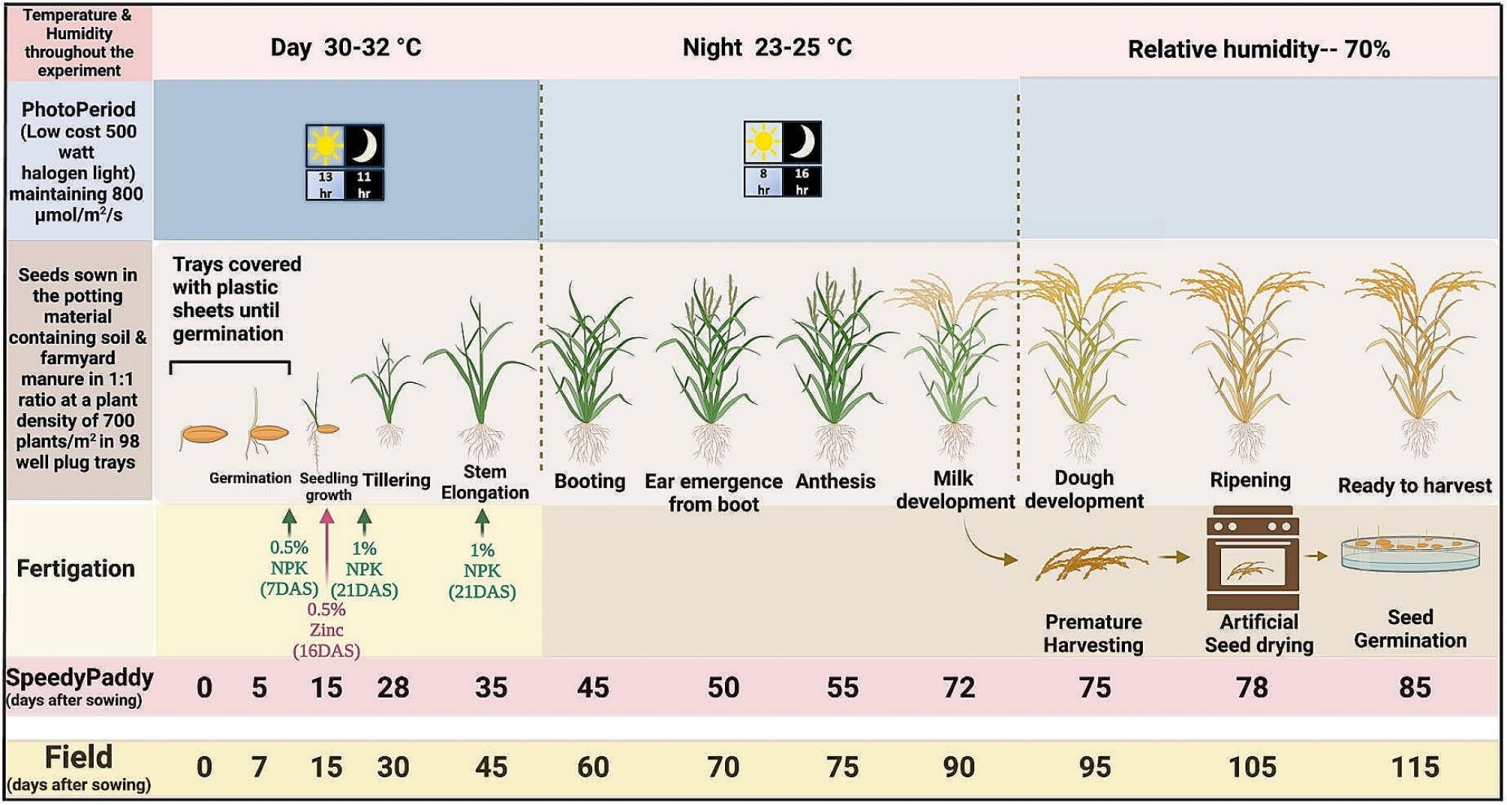
The picture illustrates the detailed compilation on the final optimized SpeedyPaddy protocol

The details on the final optimized SpeedyPaddy protocol are presented in Fig. 7. Our final optimized cost-effective SpeedyPaddy protocol with available infrastructure, demonstrates a remarkable impact of accelerated breeding in rice crop research. The standardized protocol was validated to expedite crossing, marker development, trait-based phenotyping for anaerobic germination and germination from deeper depth, mapping of genes/QTLs and advancement of generations activities. The breeding lines developed using SpeedyPaddy protocol reached to multilocation evaluation trials within 2 years instead of the usual 6 to 7 years required.

## Conclusions

The optimized SpeedyPaddy protocol offers a promising cost-effective solution to rice science community for addressing the limitations of resources, solid infrastructure, longer generation times and climate change constraints. The average saving of 2.0 to 2.6 months per breeding cycle with an integration of genomics-assisted selection, trait-based phenotyping, mapping of QTL/genes, marker development may accelerate the varietal development and release, ultimately increasing the rice genetic gain.

### Electronic supplementary material

Below is the link to the electronic supplementary material.

Supplementary Material 1

## Data Availability

The required data has been included in the supplementary information of the manuscript.

## References

[1] Ahmar S, Gill RA, Jung KH, Faheem A, Qasim MU, Mubeen M, Zhou W. Conventional and molecular techniques from simple breeding to speed breeding in crop plants: recent advances and future outlook. Int J Mol Sci. 2020;21(7):2590. 32276445 10.3390/ijms21072590 PMC7177917

[2] Anjum SA, Ashraf U, Zohaib A, Tanveer M, Naeem M, Ali I, Tabassum T, Nazir U. Growth and developmental responses of crop plants under drought stress: a review. Zemdirb-Agric. 2017;104:267–76. 10.13080/z-a.2017.104.034

[3] Atlin GN, Cairns JE, Das B. Rapid breeding and varietal replacement are critical to adaptation of cropping systems in the developing world to climate change. Global food Secur. 2017;12:31–7. 10.1016/j.gfs.2017.01.008 PMC5439485 28580238

[4] Atieno J, Li Y, Langridge P, Dowling K, Brien C, Berger B, Varshney R, Sutton T. Exploring genetic variation for salinity tolerance in chickpea using image-based phenotyping. Sci Rep. 2017;7:1300. 28465574 10.1038/s41598-017-01211-7 PMC5430978

[5] Bailey LH. Some preliminary studies of the influence of the Electric Arc lamp upon Greenhouse plants. Volume 30. Ithaca, NY, USA: Cornell University; 1891.

[6] Begna T. Speed breeding to accelerate crop improvement. Int J Agric Sc Food Technol. 2022;8:178–86. 10.17352/2455-815X.000161

[7] Bermejo C, Gatti I, Cointry E. In vitro embryo culture to shorten the breeding cycle in lentil (Lens culinaris Medik). Plant Cell Tiss Organ Cult. 2016;127:585–90. 10.1007/s11240-016-1065-7

[8] Bhatta M, Sandro P, Smith MR, Delaney O, Voss-Fels KP, Gutierrez L, Hickey LT. Need for speed: manipulating plant growth to accelerate breeding cycles. Curr Opin Plant Biol. 2021;60:101986. 33418268 10.1016/j.pbi.2020.101986

[9] Bonea D. Speed breeding and its importance for the improvement of agricultural crops. AAMC. 2022;52:59–66. 10.52846/aamc.v52i1.1314

[10] Bugbee B, Koerner G. Yield comparisons and unique characteristics of the dwarf wheat cultivar ‘USU-Apogee’. Adv Space Res. 1997;20:1891–4. 11542565 10.1016/S0273-1177(97)00856-9

[11] Cha JK, O’Connor K, Alahmad S, Lee JH, Dinglasan E, Park H, Lee SM, et al. Speed vernalization to accelerate generation advance in winter cereal crops. Mol Plant. 2022;15:1300–9. 35754174 10.1016/j.molp.2022.06.012

[12] Croser J, Mao D, Dron N, Michelmore S, McMurray L, Preston C, Bruce D, Ogbonnaya FC, Ribalta FM, Hayes J, et al. Evidence for the application of Emerging technologies to accelerate crop Improvement—A Collaborative Pipeline to Introgress Herbicide Tolerance into Chickpea. Front Plant Sci. 2021;12:779122. 34925421 10.3389/fpls.2021.779122 PMC8678039

[13] De La Fuente GN, Frei UK, Lübberstedt T. Accelerating plant breeding. Trends Plant Sci. 2013;18(12):667–72. 24080381 10.1016/j.tplants.2013.09.001

[14] Dwivedi SL, Britt AB, Tripathi L, Sharma S, Upadhyaya HD, Ortiz R. Haploids: constraints and opportunities in plant breeding. Biotechnol Adv. 2015;33(6):812–29. 26165969 10.1016/j.biotechadv.2015.07.001

[15] Ghosh S, Watson A, Gonzalez-Navarro OE, Ramirez-Gonzalez RH, Yanes L, Mendoza-Suarez M, Simmonds J, et al. Speed breeding in growth chambers and glasshouses for crop breeding and model plant research. Nat Protoc. 2018;13:2944–63. 30446746 10.1038/s41596-018-0072-z

[16] Grafius JE. Short cuts in plant breeding. Crop Sci. 1965;5:337. 10.2135/cropsci1965.0011183X000500040036x

[17] Hickey LT, Germán SE, Pereyra SA, Diaz JE, Ziems LA, Fowler RA, Platz GJ, Franckowiak JD, Dieters MJ. Speed breeding for multiple disease resistance in barley. Euphytica. 2017;213:64. 10.1007/s10681-016-1803-2

[18] Hickey LT, Hafeez N, Robinson A H. Breeding crops to feed 10 billion. Nat Biotechnol. 2019;37:744–54. 31209375 10.1038/s41587-019-0152-9

[19] Jahne F, Hahn V, Wurschum T, Leiser WL. Speed breeding short-day crops by LED-controlled light schemes. Theor Appl Genet. 2020;133:2335–42. 32399653 10.1007/s00122-020-03601-4 PMC7360641

[20] Liu BH, Wu JY, Yang SQ, Schiefelbein J, Gan YB, Xu G. Nitrate regulation of lateral root and root hair development in plants. J Exp Bot. 2020;71(15):4405–14. 31796961 10.1093/jxb/erz536 PMC7382377

[21] Lulsdorf MM, Banniza S. Rapid generation cycling of an F2 population derived from a cross between Lens culinaris Medik. And Lens ervoides (Brign.) Grande after aphanomyces root rot selection. Plant Breed. 2018;137:486–91. 10.1111/pbr.12612

[22] Mackill DJ. Rainfed lowland rice improvement. Philippines, Manila: International Rice Research Institute; 1996.

[23] Morris M, Edmeades G, Pehu E. The global need for plant breeding capacity: what roles for the public and private sectors? HortScience. 2006;41(1):30–9. 10.21273/HORTSCI.41.1.30

[24] Poehlmann JM, Sleper DA. Breeding field crops. 4th ed. City: Panima Publishing Corporation, New Dehli; 1995.

[25] Rahman MA, Quddus MR, Jahan N, Rahman MA, Sarker MRA, Hossain H, Iftekharuddaula KM. Field rapid generation advance: an effective technique for industrial scale rice breeding program. Exp. 2019;47(2):2659–70.

[26] Rana MM, Takamatsu T, Baslam M, Kaneko K, Itoh K, Harada N, Mitsui T. Salt tolerance improvement in rice through efficient SNP marker-assisted selection coupled with speed-breeding. Int J Mol Sci. 2019;20:2585. 31130712 10.3390/ijms20102585 PMC6567206

[27] Ribaut J, De Vicente M, Delannay X. Molecular breeding in developing countries: challenges and perspectives. Curr Opin Plant Biol. 2010;13:213–8. 20106715 10.1016/j.pbi.2009.12.011

[28] Rodrmguez EPB, Morante N, Salazar S, Hyde PT, Setter TL, Kulakow P, Aparicio JS, Zhang X. Flower-inducing technology facilitates speed breeding in cassava. Front Plant Sci. 2023;14:1172056. 37284728 10.3389/fpls.2023.1172056 PMC10239864

[29] Samantara K, Bohra A, Mohapatra SR, Prihatini R, Asibe F, Singh L, Reyes VP, Tiwari A, Maurya AK, Croser JS, et al. Breeding more crops in less time: a perspective on speed breeding. Biology. 2022;11:275. 35205141 10.3390/biology11020275 PMC8869642

[30] Sandhu N, Singh J, Singh G, Sethi M, Singh MP, Pruthi G, et al. Development and validation of a novel core set of KASP markers for the traits improving grain yield and adaptability of rice under direct-seeded cultivation conditions. Genomics. 2022;114(2):110269. 10.1016/j.ygeno.2022.110269 35065190

[31] Sandhu N, Yadav S, Catolos M, Cruz MTS, Kumar A. Developing climate resilient, direct-seeded, adapted multiplestress-tolerant rice applying genomics assisted breeding. Front Plant Sci. 2021;12:637488. 10.3389/fpls.2021.637488 PMC8082028 33936127

[32] Schilling S, Melzer R, Dowling CA, Shi J, Muldoon S, McCabe PF. A protocol for rapid generation cycling (speed breeding) of hemp (Cannabis sativa) for research and agriculture. Plant J. 2023;113:437–45. 36458321 10.1111/tpj.16051 PMC10108250

[33] Schoen A, Wallace S, Holbert MF, Brown-Guidera G, Harrison S, Murphy P, Sanantonio N, Van Sanford D, Boyles R, Mergoum M, et al. Reducing the generation time in winter wheat cultivars using speed breeding. Crop Sci. 2023;63:2079–90. 10.1002/csc2.20989

[34] Shimelis H, Gwata ET, Laing MD. (2019) Crop improvement for agricultural transformation in Southern Africa. In: Sikora RA, Terry ER, Vlek PLG, Chitja J, eds. Transforming Agriculture in Southern Africa. 1st ed. Routledge:97–103.

[35] Stoskopf NC, Tomes DT, Christie BR. Plant breeding: theory and practice. Boulder: Westview; 1993.

[36] Vatter T, Neuhauser B, Stetter M, Ludewig U. Regulation of length and density of Arabidopsis root hairs by ammonium and nitrate. J Plant Res. 2015;128(5):839–48. 26008190 10.1007/s10265-015-0733-8

[37] Vissenberg K, Claeijs N, Balcerowicz D, Schoenaers S, Gifford M. Hormonal regulation of root hair growth and responses to the environment in Arabidopsis. J Exp Bot. 2020;71(8):2412–27. 31993645 10.1093/jxb/eraa048 PMC7178432

[38] Watson A, Ghosh S, Williams MJ, Cuddy WS, Simmonds J, Rey MD, Asyraf Md Hatta M, et al. Speed breeding is a powerful tool to accelerate crop research and breeding. Nat Plants. 2018;4:23–9. 29292376 10.1038/s41477-017-0083-8

[39] Yao Y, Zhang P, Wang H, Lu Z, Liu CJ, Liu H, Yan GJ. How to advance up to seven generations of canola (Brassica napus L.) per annum for the production of pure line populations? Euphytica. 2016;209:113–9. 10.1007/s10681-016-1643-0

[40] Yoshida S. Fundamental of Rice Crop Science. Los Baños, Laguna, Philippines: International Rice Research Institute; 1981. p. 269.

