# Supplementary material for: SpeedyPaddy: a revolutionized cost-effective protocol for large scale offseason advancement of rice germplasm
Source: Plant Methods. 2024 Jul 20;20:109. doi: 10.1186/s13007-024-01235-x (PMC11264910; doi:10.1186/s13007-024-01235-x)
Supplement: Supplementary file 1 — Supplementary Material 1 [file 13007_2024_1235_MOESM1_ESM.docx]

**Supplementary Table 1: Detailed information on the different combinations of nutritional doses, the mode, type, and stages of application of nutrients**

| **Under Field Conditions (days)** | **Speed Breeding (days)** | **Stages** | **Sub-stages** | **Treatment 1 (Foliar Spray)** | **Treatment 2 (Fertigation)** | **Treatment 3 (Fertigation)** | **Treatment 4 (Fertigation)** | **Treatment 5 (Fertigation)** | **Treatment 6 (Fertigation)** |
| --- | --- | --- | --- | --- | --- | --- | --- | --- | --- |
| 7 | 5 | Germination | Coleoptile emerged from the seed | - | - | - | - | - | - |
| 14 | 7-8 | Seedling growth | The first leaf emerged | 1% NPK | 1% NPK | 5% MS Basal Medium | 1% NPK | 0.5% NPK | 0.5% NPK |
| 21 | 15-18 | Seedling growth | Eight leaves emerged | 1% FeSO_4_ (15^th^ day) and 1% Zn (18^th^ day) | 1% FeSO_4_ (15^th^ day) and 1% Zn (18^th^ day) | 10% MS Basal Medium | 1% Zn (15^th^ day) | 0.5% Zn (15^th^ day), 0.5% NPK (18^th^ day) | 0.5% Zn (15^th^ day), 0.5% NPK (18^th^ day) |
| 28 | 22 | Tillering | The main stem and three tillers | 1% FeSO_4_ | 1% FeSO_4_ | 5% MS Basal Medium | 1%NPK | - | - |
| 35 | 28 | Stem elongation | First node detectable | - | - | - | 1% Zn | 1%NPK | 1%NPK |
| 42 | 32 | Stem elongation | Flag leaf just visible | 1%NPK, 1% Zn | 1%NPK, 1% Zn | 10% MS Basal Medium | 1%NPK | - | - |
| 49 | 40 | Booting | Boots just visible swollen | 1%NPK | 1%NPK | - | - | - | - |
| 56 | 45 | Booting | First awns visible | - | - | - | 1%NPK | 1%NPK | - |
| 63 | 50 | Ear emergence from boot | Ear half emerged | - | - | - | - | - | - |
| 70 | 55 | Anthesis (flowering) | Beginning of anthesis | - | - | - | - | - | - |
| 77 | 60-62 | Anthesis (flowering) | Anthesis complete | - | - | - | - | - | - |
| 84 | 68 | Milk development | Early milk | - | - | - | - | - | - |
| 91 | 72 | Dough development | Early dough | - | - | - | - | - | - |
| 98 | 80 | Dough development | Hard dough | - | - | - | - | - | - |
| 105 |  | Ripening | Seed dormant | - | - | - | - | - | - |
| 112 |  | Ripening | Secondary dormancy lost | - | - | - | - | - | - |

**Supplementary Table 2: Detailed information on the different light source and photoperiod used for the Optimization of light source and photoperiod in SpeedyPaddy**

|  |  | **Photoperiod** | | |
| --- | --- | --- | --- | --- |
| **Treatment** | **Source** | **Seedling stage (light/dark)** | **Vegetative stage (light/dark)** | **Reproductive stage (light/dark)** |
| Treatment 1 (T1) | Full spectrum artificial light (PPFD of ~750–800 μmol/m^2^/s) | 8h/16h | 8h/16h | 8h/16h |
| Treatment 2 (T2) | Full spectrum artificial light (PPFD of ~750–800 μmol/m^2^/s) | 13h/11h | 13h/11h | 8h/16h |
| Treatment 3 (T3) | No artificial light | Natural spectrum | | |
| Treatment 4 (T4) | Halogen bulbs (B:G:R:FR -7.0:27.6:65.4:89.2) | 13h/11h | 13h/11h | 13h/11h |
| Treatment 5 (T5) | Halogen bulbs (B:G:R:FR -7.0:27.6:65.4:89.2) | 13h/11h | 13h/11h | 8h/16h |

**Supplementary Table 3: Detailed information on different combinations of soil (S), farmyard manure (FYM) and cocopeat (C) were tested on varieties of different durations**

| **Treatment** | **Potting mixture** | **Days to germination** |
| --- | --- | --- |
| Treatment 1 | S-40: FYM-40: C-20 | 8 days |
| Treatment 2 | S-50: FYM-25: C-25 | 9 days |
| Treatment 3 | S-50: FYM-50: C-0 | 5 days |
| Treatment 4 | S-30: FYM-70: C-0 | 5 days |
| Treatment 5 | S-20: FYM-60: C-20 | 7 days |
| Treatment 6 | S-60: FYM-0: C-40 | 12 days |
| Treatment 7 | S-50: FYM-40: C-10 | 6 days |
| Treatment 8 | S-90: FYM-10: C-0 | 10 days |

**Supplementary Table 4: Detailed information on effect of different plant density on mean plant height of varieties of different durations.**

|  |  | **Plant height (cm)** | | | | | | | |
| --- | --- | --- | --- | --- | --- | --- | --- | --- | --- |
|  |  | **Early duration** | | | **Medium duration** | | | **Late duration** | |
| **Treatment** |  | **PR126** | **PB1509** | **MTU1010** | **PR121** | **PR128** | **PR129** | **Swarna** | **Sambha Mahsuri** |
| **Treatment 1 (T1)** | **Pot** | 97 | 100 | 88 | 90 | 90 | 90 | 85 | 85 |
| **Treatment 2 (T2)** | **21 wells tray** | 95 | 92 | 85 | 88 | 90 | 89 | 88 | 85 |
| **Treatment 3 (T3)** | **50 wells tray** | 91.8 | 88.6 | 89.0 | 92.0 | 87.1 | 87.1 | 82.4 | 84.6 |
| **Treatment 4 (T4)** | **98 wells tray** | 55.0 | 52.0 | 55.0 | 57.0 | 58.0 | 56.0 | 55.0 | 55.0 |

**Supplementary Table 5: Detailed information on effect of different plant density on mean days to flowering (days) of varieties of different durations.**

|  |  | **Days to flowering (days)** | | | | | | | |
| --- | --- | --- | --- | --- | --- | --- | --- | --- | --- |
|  |  | **Early duration** | | | **Medium duration** | | | **Late duration** | |
| **Treatment** |  | **PR126** | **PB1509** | **MTU1010** | **PR121** | **PR128** | **PR129** | **Swarna** | **Sambha Mahsuri** |
| **Treatment 1 (T1)** | **Pot** | 75 | 75 | 75 | 85 | 86 | 85 | 85 | 90 |
| **Treatment 2 (T2)** | **21 wells tray** | 70 | 72 | 72 | 80 | 82 | 82 | 82 | 85 |
| **Treatment 3 (T3)** | **50 wells tray** | 63 | 68 | 69 | 73 | 73 | 72 | 73 | 75 |
| **Treatment 4 (T4)** | **98 wells tray** | 56 | 56 | 56 | 55 | 55 | 58 | 57 | 58 |

**Supplementary Table 6: Detailed information on effect of different nutrient treatments on mean plant height (cm) of varieties of different durations.**

| **Variety** | **Treatment** | **Minimum (cm)** | **Maximum (cm)** | **Mean (cm)** | **Standard error_Mean** |
| --- | --- | --- | --- | --- | --- |
| PR126 | Treatment 2 (T2) | 63 | 111 | 93 | 2.28 |
| PB1509 | Treatment 2 (T2) | 79 | 99 | 88 | 1.13 |
| MTU1010 | Treatment 2 (T2) | 78 | 103 | 87 | 1.26 |
| PR121 | Treatment 2 (T2) | 75 | 106 | 93 | 1.19 |
| PR128 | Treatment 2 (T2) | 81 | 100 | 88 | 1 |
| PR129 | Treatment 2 (T2) | 82 | 101 | 89 | 1 |
| PR130 | Treatment 2 (T2) | 86 | 107 | 97 | 0.9 |
| Swarna | Treatment 2 (T2) | 86 | 107 | 98 | 0.85 |
| Samba Mahsuri | Treatment 2 (T2) | 62 | 110 | 92 | 2.28 |
| PR126 | Treatment 4 (T4) | 80 | 100 | 89 | 1.16 |
| PB1509 | Treatment 4 (T4) | 80 | 105 | 89 | 1.26 |
| MTU1010 | Treatment 4 (T4) | 74 | 105 | 92 | 1.19 |
| PR121 | Treatment 4 (T4) | 80 | 99 | 87 | 1 |
| PR128 | Treatment 4 (T4) | 80 | 99 | 87 | 1 |
| PR129 | Treatment 4 (T4) | 84 | 105 | 95 | 0.9 |
| PR130 | Treatment 4 (T4) | 84 | 105 | 96 | 0.85 |
| Swarna | Treatment 4 (T4) | 56 | 74 | 65 | 0.84 |
| Samba Mahsuri | Treatment 4 (T4) | 55 | 75 | 64 | 1.16 |
| PR126 | Treatment 5 (T5) | 55 | 80 | 64 | 1.26 |
| PB1509 | Treatment 5 (T5) | 49 | 80 | 67 | 1.19 |
| MTU1010 | Treatment 5 (T5) | 55 | 74 | 62 | 1 |
| PR121 | Treatment 5 (T5) | 55 | 74 | 62 | 1 |
| PR128 | Treatment 5 (T5) | 59 | 80 | 70 | 0.9 |
| PR129 | Treatment 5 (T5) | 59 | 80 | 71 | 0.85 |
| PR130 | Treatment 5 (T5) | 42 | 60 | 51 | 0.9 |
| Swarna | Treatment 5 (T5) | 42 | 63 | 51 | 1 |
| Samba Mahsuri | Treatment 5 (T5) | 41 | 65 | 54 | 1.32 |
| PR126 | Treatment 6 (T6) | 48 | 77 | 59 | 1.26 |
| PB1509 | Treatment 6 (T6) | 51 | 69 | 59 | 0.83 |
| MTU1010 | Treatment 6 (T6) | 43 | 65 | 55 | 1.22 |
| PR121 | Treatment 6 (T6) | 45 | 66 | 56 | 0.9 |
| PR128 | Treatment 6 (T6) | 45 | 66 | 57 | 0.85 |

For the details on treatments please see Supplementary Table 1

Treatment 1 (T1) and Treatment 3 (T3): Plant does not survive

**Supplementary Table 7: Detailed information on effect of different nutrient treatments on mean days to flowering (days) of varieties of different durations.**

| **Variety** | **Treatment** | **Minimum (days)** | **Maximum (days)** | **Mean** | **Standard Error_Mean** | **Coefficient of variations (%)** |
| --- | --- | --- | --- | --- | --- | --- |
| PR126 | Treatment 2 (T2) | 75 | 80 | 78 | 1.453 | 3.24 |
| PB1509 | Treatment 2 (T2) | 75 | 80 | 77 | 1.453 | 3.25 |
| MTU1010 | Treatment 2 (T2) | 75 | 81 | 79 | 1.856 | 4.09 |
| PR121 | Treatment 2 (T2) | 82 | 88 | 85 | 1.732 | 3.53 |
| PR128 | Treatment 2 (T2) | 85 | 88 | 86 | 0.882 | 1.77 |
| PR129 | Treatment 2 (T2) | 84 | 88 | 86 | 1.202 | 2.43 |
| PR130 | Treatment 2 (T2) | 84 | 88 | 86 | 1.202 | 2.43 |
| Swarna | Treatment 2 (T2) | 90 | 94 | 92 | 1.155 | 2.17 |
| Samba Mahsuri | Treatment 2 (T2) | 90 | 95 | 93 | 1.528 | 2.84 |
| PR126 | Treatment 4 (T4) | 70 | 75 | 72 | 1.453 | 3.48 |
| PB1509 | Treatment 4 (T4) | 72 | 75 | 73 | 1.000 | 2.37 |
| MTU1010 | Treatment 4 (T4) | 72 | 78 | 75 | 1.732 | 4.00 |
| PR121 | Treatment 4 (T4) | 80 | 85 | 82 | 1.453 | 3.06 |
| PR128 | Treatment 4 (T4) | 82 | 88 | 84 | 2.000 | 4.12 |
| PR129 | Treatment 4 (T4) | 82 | 85 | 84 | 0.882 | 1.83 |
| PR130 | Treatment 4 (T4) | 82 | 88 | 85 | 1.764 | 3.61 |
| Swarna | Treatment 4 (T4) | 85 | 88 | 87 | 1.000 | 1.99 |
| Samba Mahsuri | Treatment 4 (T4) | 84 | 88 | 87 | 1.333 | 2.66 |
| PR126 | Treatment 5 (T5) | 63 | 68 | 65 | 1.453 | 3.85 |
| PB1509 | Treatment 5 (T5) | 65 | 68 | 67 | 1.000 | 2.59 |
| MTU1010 | Treatment 5 (T5) | 65 | 70 | 68 | 1.528 | 3.89 |
| PR121 | Treatment 5 (T5) | 70 | 73 | 72 | 0.882 | 2.13 |
| PR128 | Treatment 5 (T5) | 72 | 75 | 73 | 0.882 | 2.08 |
| PR129 | Treatment 5 (T5) | 68 | 72 | 71 | 1.333 | 3.27 |
| PR130 | Treatment 5 (T5) | 68 | 73 | 70 | 1.453 | 3.58 |
| Swarna | Treatment 5 (T5) | 72 | 75 | 74 | 1.000 | 2.34 |
| Samba Mahsuri | Treatment 5 (T5) | 74 | 75 | 75 | 0.333 | 0.77 |
| PR126 | Treatment 6 (T6) | 56 | 58 | 57 | 0.667 | 2.01 |
| PB1509 | Treatment 6 (T6) | 56 | 60 | 58 | 1.155 | 3.45 |
| MTU1010 | Treatment 6 (T6) | 56 | 60 | 58 | 1.155 | 3.45 |
| PR121 | Treatment 6 (T6) | 55 | 60 | 57 | 1.667 | 5.09 |
| PR128 | Treatment 6 (T6) | 55 | 58 | 56 | 1.000 | 3.09 |
| PR129 | Treatment 6 (T6) | 58 | 60 | 59 | 0.667 | 1.95 |
| PR130 | Treatment 6 (T6) | 57 | 60 | 59 | 1.000 | 2.94 |
| Swarna | Treatment 6 (T6) | 58 | 62 | 60 | 1.155 | 3.33 |
| Samba Mahsuri | Treatment 6 (T6) | 60 | 62 | 61 | 0.667 | 1.90 |

For the details on treatments please see Supplementary Table 1

Treatment 1 (T1) and Treatment 3 (T3): Plant does not survive

**Supplementary Table 8: Detailed information on effect of different source and photoperiod of light on mean days to flowering (days) of varieties of different durations**

| **Treatment** | **Early duration** | | | **Medium duration** | | | **Late duration** | |
| --- | --- | --- | --- | --- | --- | --- | --- | --- |
|  | **PR126** | **PB1509** | **MTU1010** | **PR121** | **PR128** | **PR129** | **Swarna** | **Sambha** **Mahsuri** |
| Treatment 1 (T1) | 78 | 77 | 79 | 85 | 86 | 86 | 92 | 93 |
| Treatment 2 (T2) | 58 | 58 | 59 | 58 | 57 | 60 | 60 | 60 |
| Treatment 3 (T3) | - | - | - | - | - | - | - | - |
| Treatment 4 (T4) | 65 | 67 | 68 | 72 | 73 | 71 | 74 | 75 |
| Treatment 5 (T5) | 57 | 58 | 58 | 57 | 56 | 59 | 60 | 61 |

For the details on treatments please see Supplementary Table 1

**Supplementary Table 9: The estimated cost to run one breeding cycle of three months in SpeedyPaddy**

| **Requirements** | **Description** | **Cost per unit (Rs.)** | **Quantity** | **Total Cost (Rs)** | **Total Cost $** |
| --- | --- | --- | --- | --- | --- |
| Light | 500-watt halogen bulb | 100 | 20 | 2000 | 25 |
| Heaters | 300-watt coiled heaters | 5000 | 2 | 10000 | 125 |
| Nutrients | 14N:14P:14K, Zn | 500 | 2 | 1000 | 12.5 |
| Electricity consumption | heaters: 2 (1500 watt each; Halogen bulb: 20 (500 watt each), Humidifier (260 watt) | 10 | 12265 | 122650 | 1533.125 |
| Labor cost | Labor cost | 500 | 90 | 45000 | 562.5 |
| Miscellaneous expenses | Plastic crate* | 300 | 160 | 48000 | 600 |
|  | Plug trays | 10 | 160 | 1600 | 20 |
| Maintenance cost | including fixing of electricity, halogen bulb, heaters, humidifiers etc. | 5000 | 1 | 5000 | 62.5 |
| Total Cost |  |  |  | 209850 | 2940.625 |
|  |  |  |  |  |  |
| ***one time cost** |  |  |  |  |  |
| **$= Rs 80** |  |  |  |  |  |

**Supplementary Table 10. Optimization of premature seed germination in SpeedyPaddy**

| Treatments | Percentage germination (%) |
| --- | --- |
| Treatment 1 (T1): 100 ppm GA_3_ | 70 |
| Treatment 2 (T2): 2% CaCl_2_ | 65 |
| Treatment 3 (T3): 1% KH_2_PO_4_ | 20 |
| Treatment 4 (T4): 40 ppm Na2SeO_3_ | 29 |

**Supplementary Table 11. The mean saving of days in the early, medium, and late duration tested rice varieties in SpeedyPaddy compared to field conditions**

|  | **Variety** | **Days to flowering_**  **Field (days)** | **Maturity_**  **Field (days)** | **Days to flowering _SpeedyPaddy (days)** | **Maturity_**  **SpeedyPaddy (days)** | **Total days saved (days)** | **No. of generations/year** |
| --- | --- | --- | --- | --- | --- | --- | --- |
| Early duration | PR126 | 88 | 123 | 55 | 70 | 53 | 5.2 |
|  | PB1509 | 90 | 120 | 56 | 71 | 49 | 5.14 |
|  | MTU1010 | 90 | 120 | 55 | 70 | 50 | 5.2 |
| Medium duration | PR121 | 100 | 130 | 53 | 68 | 62 | 5.3 |
|  | PR128 | 101 | 131 | 55 | 70 | 61 | 5.2 |
|  | PR129 | 100 | 130 | 57 | 72 | 58 | 5 |
| Late duration | Swarna | 115 | 150 | 58 | 73 | 77 | 5 |
|  | Sambha Mahsuri | 115 | 150 | 60 | 75 | 75 | 4.8 |
